# Can we find the missing men in clinics? Clinic attendance by sex and HIV status in rural South Africa

**DOI:** 10.12688/wellcomeopenres.16702.1

**Published:** 2021-07-02

**Authors:** Safiyya Randera-Rees, Wende Clarence Safari, Dickman Gareta, Kobus Herbst, Kathy Baisley, Alison D. Grant

**Affiliations:** 1 Africa Health Research Institute, KwaZulu Natal, South Africa; 2 DSI-MRC, South African Population Research Infrastructure Network, KwaZulu Natal, South Africa; 3 Faculty of Epidemiology and Population Health, London School of Hygiene and Tropical Medicine, London, WC1E 7HT, UK; 4 School of Laboratory Medicine & Medical Sciences, College of Health Sciences, University of KwaZulu-Natal, Durban, South Africa; 5 School of Public Health, University of the Witwatersrand, Johannesburg, South Africa; 6 London School of Hygiene & Tropical Medicine, London, WC1E 7HT, UK

**Keywords:** Tuberculosis, Africa, Active Case Finding, Primary Health Care

## Abstract

**Background:** HIV-negative men are over-represented in tuberculosis (TB) prevalence surveys including the first South African national TB prevalence survey in 2018. Traditionally, TB screening is focused in clinics. We aimed to determine the frequency of primary healthcare clinic (PHC) attendance among HIV-negative men in a TB-prevalent setting.

**Methods:** Since January 2017, PHC attendees in a rural South African demographic surveillance area (DSA) were asked their reason for attendance. HIV status was defined as positive if tested positive in a DSA sero-survey or attended clinic for HIV care; negative if tested negative between January 2014—December 2017 and no HIV-related visits; and HIV-unknown otherwise.

**Results:** Among 67124 DSA residents (≥15 years), 27038 (40.3%) were men; 14196 (21.2%) were classified HIV-positive, 18892 (28.1%) HIV-negative and 34036 (50.7%) HIV-unknown. Between April 2017 and March 2018, 24382/67124 (36.3%, 95% confidence interval [CI] 36.0–36.7) adults made ≥1 PHC visit, comprising 9805/40086 (24.5%, 95%CI 23.6–25.3) of HIV-negative or unknown women and 3440/27038 (12.7%, 95%CI 11.6–13.8) of HIV-negative or unknown men. Overall, HIV care accounted for 37556/88109 (42.6%) of adult PHC visits.

**Conclusion:** In this rural population, HIV-negative and -unknown men rarely attend PHCs. Improving TB screening in clinics may not reach a key population with respect to undiagnosed TB. Additional strategies are needed to diagnose and treat TB earlier.

## Introduction

Based on data from 2013, South Africa was estimated to have 160000 “missing” people with tuberculosis (TB), that is individuals with active TB disease who were not on treatment ^1^, who may contribute to continuing transmission. South Africa is committed to finding the missing 160000 ^1^; however, how best to do this is uncertain.

The World Health Organization (WHO) has traditionally recommended active case finding for TB among individuals attending health facilities ^2^. However, this approach will only reduce transmission if people with undiagnosed, infectious TB are identified at health facilities and start appropriate treatment early, and therefore reduce their duration of infectiousness. This is particularly a concern for men, who are generally perceived to attend health care facilities less often than women ^3, 4^.

National surveys in Tanzania, Rwanda, Zambia and Kenya have found that TB prevalence was significantly higher in men than women ^5– 7^. In addition, in Zambia and Kenya (where the prevalence surveys offered HIV testing as well as TB screening) over 80% people with undiagnosed active TB were HIV-negative or HIV-unknown. In South Africa the first national TB prevalence survey took place in 2018. Among the survey participants who reported at least one TB symptom, more men (71.3%) than women (63.4%) did not seek care ^8^. TB prevalence was higher in men, with a prevalence almost 1.6 times that of women ^8^. 77% of participants who screened positive for TB were HIV negative or unknown ^8^. It is therefore likely that a priority group among the “missing 160000” are men with negative or unknown HIV status ^8^.

The aim of this study was to describe the frequency of primary healthcare clinic (PHC) attendance by sex and HIV status in 11 clinics in rural KwaZulu-Natal, South Africa.

## Methods

### Study area and population

The study was conducted in the Africa Health Research Institute (AHRI) demographic surveillance area (DSA), in uMkhanyakude district, KwaZulu-Natal, South Africa. The AHRI DSA covers 845km ^2^, with approximately 25000 homesteads and over 60000 residents aged 15 years or above. AHRI has undertaken population-based demographic surveillance since 2000 with annual HIV sero-surveys since 2003. This district had an annual notification rate of all TB cases of 394 per 100,000 population in 2018 and 64.4% of those notified for TB were HIV positive ^9^. In addition, in 2017-8, 9.9% of Xpert MTB/RIF results from uMkhanyakude showed rifampicin resistance, among the highest prevalence in the country ^9^.

The population of interest in our analysis were all individuals over the age of 15 years who were a resident member (defined as intending to spend the majority of nights at a household within the study area) of a household in the AHRI DSA on 1st July 2017.

### Data collection

Since January 2017, individuals who sought health care at any one of the 11 PHC’s serving the AHRI DSA on weekdays between 7am and 7pm have been registered by a member of AHRI staff, and their self-reported reason for attending clinic recorded, using an electronic system known as ClinicLink ^10^. For this analysis, we used ClinicLink data to determine the number of PHC visits made by AHRI DSA residents between 1 April 2017 and 31 March 2018. Visit data from AHRI DSA residents were retrospectively linked to their demographic surveillance data and HIV sero-survey data. 

### Case definitions

Participants were considered to be HIV negative if they tested negative in a sero-survey between 1st January 2014 and 31st December 2017 and had no HIV-related PHC visits recorded in ClinicLink; HIV-positive if they tested HIV-positive in a DSA sero-survey or had an HIV-related visit recorded in ClinicLink; or HIV-unknown otherwise.

### Statistical analysis

The main outcome for this analysis was the proportion of DSA residents visiting PHCs between April 2017 and March 2018, stratified by sex and HIV status.

We categorized PHC visits into three subgroups: 1. HIV visits, including antiretroviral therapy (ART) start or follow-up; 2. acute visits, including family planning, minor ailments, maternity, reproductive health, circumcision, or emergency care; and 3. other chronic (non-HIV), including care for TB, diabetes or hypertension.

All data was analysed using Stata (StataCorp. 2017. *Stata Statistical Software: Release 14.1*. College Station, TX: StataCorp LLC).

### Ethical approval

The AHRI demographic surveillance system, ClinicLink study and linkage to Department of Health ART records are approved by the Biomedical Research Ethics Committee of the University of KwaZulu-Natal, South Africa (Ref: BE290/16). DSA residents give written informed consent for household demographic surveys, individual health and behaviour questionnaires, and HIV sero-surveys. Individuals attending clinics provide written informed consent to record visits.

## Results

Table 1 shows the demographic characteristics of 67124 resident adults (40086 [59.7%] women) over the age of 15 years in the AHRI DSA on 1 ^st^ July 2017. Of all residents included, 14196/67124 (21.2%) were classified as HIV-positive, 18892/67124 (28.1%) HIV-negative and 34036/67124 (50.7%) HIV-unknown. Among women, 10692/40086 (26.7%) were HIV-positive ^11^. Furthermore, among 27038 men, 3504/27038 (13.0%) were classified as HIV-positive and 16630 (61.5%) HIV-unknown. 

**Table 1.** HIV status of 67124 adults resident in the Africa Health Research Institute demographic surveillance area ^1^, stratified by sex.

Characteristics	Total	Females	Males
Total, N (row %)	67124	40086 (59.7%)	27038 (40.3)
HIV-positive ^2^, N (column %)	14196 (21.2)	10692 (26.7%)	3504 (13.0)
HIV-negative ^3^, N (column %)	18892 (28.1)	11988 (29.9%)	6904 (25.5)
HIV-unknown ^4^, N (column %)	34036 (50.7)	17406 (43.4%)	16630 (61.5)

^1^ Africa Health Research Institute (AHRI) undertakes population-based demographic surveillance in a rural district of Kwazulu Natal, South Africa with annual HIV sero-surveys. Individuals were included if they were resident in the AHRI demographic surveillance area on 1st July 2017. ^2^ Participants were considered to be HIV-positive if they tested HIV-positive in a demographic surveillance area sero-survey or had an HIV-related visit recorded. ^3^ Participants were considered to be HIV negative if they tested negative in a sero-survey between 1st January 2014 and 31st December 2017 and had no HIV-related PHC visits recorded. ^4^ Participants were considered to be HIV unknown if neither 2 nor 3 was true.

Table 2 shows the proportion of adults making one or more PHC visits (for any reason) during the study year, by sex and HIV status. Among all 67124 residents, 36.3% (95% confidence interval [CI] 36.0–36.7) visited one of the eleven PHCs serving the AHRI DSA during the study period. The median number of PHC visits (among the 24382 residents who visited a PHC) was two (range: 1–26) per person among men and three (range: 1–22) visits per person among women. Of the 67124 adult DSA residents, among HIV-positive individuals, 8554/10692 (80%, 95%CI 79.3–80.8) of HIV-positive women and 2593/3504 (73.7%, 95%CI 72.2–75.1) of HIV positive men visited a PHC for any reason at least once during the one-year period. In contrast, among the HIV-negative or HIV-unknown adult residents, 9805/29394 (33.4%, 95%CI 32.8–33.9) of women and 3340/23534 (14.6%, 95%CI 14.2–15.1) of men visited a PHC at least once during the one-year period of the study.

**Table 2.** Proportion of adults resident in the Africa Health Research Institute demographic surveillance area ^1^ who attended a primary healthcare clinic between April 2017 and March 2018, stratified by sex and HIV status.

Sex	HIV-positive			HIV-negative/HIV-unknown			All		
	n ^2^	N ^3^	%	n ^2^	N ^3^	%	n ^2^	N ^3^	%
			(CI)			(CI)			(CI)
Females	8555	10692	80.0 (79.3,80.8)	9805	29394	33.4 (32.8,33.9)	18360	40086	45.8 (45.3,46.3)
Males	2582	3504	73.7 (72.2,75.1)	3440	23534	14.6 (14.2,15.1)	6022	27038	22.2 (21.8,22.8)
Total	11137	14196	78.5 (77.8,79.1)	13145	52928	24.8 (24.5,25.2)	24382	67124	36.3 (36.0,36.7)

^1^ Africa Health Research Institute (AHRI) undertakes population-based demographic surveillance in a rural district of Kwazulu Natal, South Africa with annual HIV sero-surveys. Individuals were included if they were resident in the AHRI demographic surveillance area on 1st July 2017. ^2^ n represents the number of resident adults making ≥1 primary healthcare clinic visit (for any reason) in the study year. ^3^ N represents number of resident adults in the Africa Health Research Institute Demographic Surveillance Area’s population for each category.

AHRI adult residents made a total of 88109 visits to the 11 PHCs during the study period ( Table 3). Of the total number of PHC visits, HIV care accounted for 37556/88109 (42.6%), acute conditions for 31147/88109 (35.4%) while other chronic care accounted for 19406/88109 (22.0%). Excluding visits for antenatal and paediatric care, there were more PHC visits by women compared to men regardless of HIV status among all visit categories.

**Table 3.** Number and type of primary healthcare visits made by adults resident in the Africa Health Research Institute demographic surveillance area ^1^ during the study year by sex (N=88109).

Sex	HIV care visits		Acute care visits		Other chronic care visits		Total	
	n ^2^	% ^3^	n ^2^	% ^3^	n	% ^3^	n ^2^	% ^3^
Females	29384	33.3	25303	28.7	15000	17.0	69687	79.1
Males	8172	9.3	5844	6.6	4406	5.0	18422	20.9
Total	37556	42.6	31147	35.4	19406	22.0	88109	100

^1^ Africa Health Research Institute (AHRI) undertakes population-based demographic surveillance in a rural district of KwaZulu Natal, South Africa with annual HIV sero-surveys. Individuals were included if they were resident in the AHRI demographic surveillance area on 1st July 2017. ^2^ n represents the number of visits. ^3^ The percentages represent n/N, where N is the total visits made by all adults during study period.

## Discussion

By nesting this study within a demographic surveillance area, we have been able, for the first time, to quantify PHC visits based on a population denominator and stratified by HIV status. As anticipated, we found that men who are HIV-negative or with unknown HIV status rarely visit PHCs for any reason. Reducing TB transmission requires that people with active TB are identified and start effective treatment early, in order to reduce their duration of infectiousness. The traditional approach of "passive case finding" depends on people who are symptomatic with active TB seeking care in clinics and being successfully diagnosed and treated ^12^. The rationale for "intensified case finding" among clinic attendees is that it is far more efficient for health workers to screen for TB among people attending clinics than in the general population ^2^. However, if people with TB have relatively mild or intermittent symptoms ^13^, particularly if they are HIV-negative or unaware of their status, they may not prioritise seeking care. Our data show that HIV-negative or -unknown men rarely visit PHCs, and therefore attempts to reduce the duration of infectiousness of HIV-negative men with active TB will need to reach outside health facilities. These data illustrate that HIV care has become a dominant reason for PHC attendance among adults in this setting of very high HIV prevalence, accounting for nearly half of all daytime visits by adults. This finding is unlikely to be generalisable to settings where HIV prevalence is less high. However, as we move towards 90:90:90 and then 95:95:95 HIV care cascade targets, and HIV prevalence increases as ART prolongs survival, PHCs in many settings will need to provide care for increasing numbers of people on ART. Our data underline the importance of efforts to simplify HIV care by reducing visit frequency, and strengthening systems to support further decentralisation of ART delivery to community level ^14^.

Several studies both in South Africa and the wider African region have quantified reasons for visits to health facilities, but we are not aware of similar studies based in demographic surveillance areas or other settings with a population denominator and comprehensive data on HIV status ^15^.

A study in the Western Cape in 2016 used home-based surveys to determine if respondents had attended a PHC facility in the past six months. Similar to our finding, they found that men were less likely to attend clinic that women ^16^. Furthermore fewer than 20% of all men aged 18–25 years, or men aged 26–45 who attended bars, attended a clinic ^16^.

A study in rural Tanzania in 2019 examined the quality of outpatient care of 2002 women seen in PHCs by asking women about their most recent outpatient experience in a household survey in 2002 ^17^. The study found that the most common reasons for seeking care were fever or malaria (33.9%), vaccination of infants (33.6%) and non-emergency check-ups (13.4%) ^17^. This difference is expected given that malaria is rare in South Africa ^17^ with 9478 malaria cases and 76 malaria deaths in 2017 ^18^, whereas Tanzania had 4241364 malaria cases and 6313 deaths in 2015 ^19^.

In South Africa, a study in 2010 looked specifically at chronic non-communicable diseases in PHC facilities in the Western Cape, North West, Northern Cape and Limpopo provinces in South Africa. Health care workers recorded the age and gender of each patient and the reasons for each encounter. They found that hypertension was the commonest non-communicable disease (NCD) diagnosis encountered (13.1%), followed by type 2 diabetes (3.9%) ^20^. Only 1.1% of respondents with an NCD also had HIV by self-report ^20^. Women accounted for 12526 (66.6%) of consultations and men for 6288 (33.4%) ^20^. However, this study was focused on multimorbidity and did not include HIV care as an independent reason for clinic attendance. Furthermore, it did not consider acute care as a primary reason for consultation.

In the past 30 years, the number of individuals receiving care for HIV in clinics has grown significantly with improved and comprehensive ART treatment services ^21^. Given our finding that HIV-negative men rarely visit PHCs, improved coverage of TB screening in PHCs is likely to be inadequate to achieve earlier TB diagnosis and treatment among this important group. Additional research is needed to determine how to promote early diagnosis of TB among men. Facilitators to improve men’s access and utilisation of PHC clinics including improved models of male-centred care, and novel approaches need to be explored ^22^. Barriers that prevent men testing and accessing health services, including stigma, need to be better understood ^16, 22^. Novel approaches are necessary to access and provide TB screening services to men ^22^. Innovative strategies are needed to access this notoriously hard to reach key population, interrupt TB transmission and find the “missing 160000”.

Limitations of this analysis include that clinic attendance was only captured in the eleven PHCs serving the AHRI DSA, therefore any visits by residents in our study population to more distant clinics, hospitals and private institutions would have been missed. The number of PHC visits we recorded should therefore be regarded as a minimum estimate. In addition, no data capture occurred at night and over weekends, which means that some acutely ill people, and those seeking care for trauma will have been missed, particularly at the one PHC which is open 24 hours a day. However, it might be difficult to implement TB screening in people who are attending for acute or trauma care and so this is unlikely to substantially change our conclusions.

Some misclassification of HIV status is likely. Participants were considered to be HIV negative if they tested negative between the 1 ^st^ of January 2014 and the 31st December 2017 and had no evidence of HIV-positive status. This may have resulted in misclassification of HIV-negative people as HIV-unknown. We grouped HIV positive and unknown together so this does not affect our conclusions. A participant was considered to be HIV-positive if the last HIV test recorded by AHRI’s DSA was positive or if they have had any HIV-related PHC visit in the DSA. Therefore, anyone who tested HIV positive outside the DSA and did not attend for HIV care in the eleven DSA clinics or became HIV-positive after testing negative will have been misclassified as HIV-negative or -unknown ^23^.

## Conclusion

In rural South Africa, only 15% HIV-negative or unknown men attended a PHC for any reason during the year this research was conducted. Therefore, improving TB screening in clinics as an isolated strategy will not reach a key population with respect to undiagnosed TB. Additional strategies are likely to be needed to diagnose and treat TB earlier, particularly in men.

## Data availability

### Underlying data

AHRI Data Repository: Clinic attendance by sex and HIV status in rural South Africa. https://doi.org/10.23664/AHRI.CLINIC.VISITS.CODE.2021 ^11^.

Data are available under the terms of the Creative Commons Attribution 4.0 International license (CC-BY 4.0).
