# Supplementary material for: Can we find the missing men in clinics? Clinic attendance by sex and HIV status in rural South Africa
Source: Wellcome Open Res. 2023 Aug 21;6:169. Originally published 2021 Jul 2. [Version 2] doi: 10.12688/wellcomeopenres.16702.2 (PMC10521066; doi:10.12688/wellcomeopenres.16702.2)
Supplement: Peer review history

# Reviewer report: Reviewer response for version 2

**DOI:** 10.21956/wellcomeopenres.21810.r65672

**Published:** 2023-09-12

**Authors:** Sizulu Moyo

**Affiliations:** 1 University of Cape Town, Cape Town, South Africa; 2 Human and Social Capabilities Division, Human Sciences Research Council, Cape Town, South Africa

The revisions are appropriate.

Is the work clearly and accurately presented and does it cite the current literature?

Partly

If applicable, is the statistical analysis and its interpretation appropriate?

Yes

Are all the source data underlying the results available to ensure full reproducibility?

Yes

Is the study design appropriate and is the work technically sound?

Yes

Are the conclusions drawn adequately supported by the results?

Yes

Are sufficient details of methods and analysis provided to allow replication by others?

Yes

Reviewer Expertise:

Public health

I confirm that I have read this submission and believe that I have an appropriate level of expertise to confirm that it is of an acceptable scientific standard.

# Reviewer report: Reviewer response for version 1

**DOI:** 10.21956/wellcomeopenres.18417.r51640

**Published:** 2022-08-12

**Authors:** Sizulu Moyo

**Affiliations:** 1 University of Cape Town, Cape Town, South Africa; 2 Human and Social Capabilities Division, Human Sciences Research Council, Cape Town, South Africa

Thank you for this manuscript that casts a needed spotlight on a population group that requires attention in efforts to address the TB burden. The manuscript is well written.

 Did the authors analyze the data by age group? Stratification by age will also be helpful since it likely affects the decision and ability to attend PHC facilities:- for example the SA TB prevalence survey found the largest prevalence to notification ratio in younger people (15-24 years) and older people 65 years+. This may also be true by sex and could direct targeting and differentiation of interventions for different segments of the male population.

 Did the authors consider separating the HIV-ve and HIV status-unknown groups? Those whose status is unknown group could be different from those LWH and those HIV-ve since they have never accessed testing and could require further and different attention and interventions - did they even attend PHCs at all even for chronic conditions? 

 Please add more literature including some of the more recent literature that explores men’s views on TB and healthcare facility attendance. Chikovore J *et al *have published extensively on this subject (see reference list for example) ^1^. Issues around male attendance at health facilities are also documented in the SA CRG report: *South African Community Rights and Gender Assessment: Exploring the impact of gender, key population membership, and the legal environment on TB vulnerability, treatment access, and quality of care* ^2^.

Is the work clearly and accurately presented and does it cite the current literature?

Partly

If applicable, is the statistical analysis and its interpretation appropriate?

Yes

Are all the source data underlying the results available to ensure full reproducibility?

Yes

Is the study design appropriate and is the work technically sound?

Yes

Are the conclusions drawn adequately supported by the results?

Yes

Are sufficient details of methods and analysis provided to allow replication by others?

Yes

Reviewer Expertise:

Public health

I confirm that I have read this submission and believe that I have an appropriate level of expertise to confirm that it is of an acceptable scientific standard, however I have significant reservations, as outlined above.

## References

[1] : Missing men with tuberculosis: the need to address structural influences and implement targeted and multidimensional interventions. *BMJ Glob Health* .5(5) : 10.1136/bmjgh-2019-002255 10.1136/bmjgh-2019-002255 32371568PMC7223013

[2] : South African Community Rights and Gender Assessment: Exploring the impact of gender, key population membership, and the legal environment on TB vulnerability, treatment access, and quality of care. *Stop TB Partnership* .2019; Reference source

# Reviewer report: Reviewer response for version 1

**DOI:** 10.21956/wellcomeopenres.18417.r50585

**Published:** 2022-06-08

**Authors:** Ellen M. H. Mitchell

**Affiliations:** 1 Department of Public Health, Institute for Tropical Medicine, Antwerp, Belgium

It is conventional wisdom to assert that men do not seek health care at the same frequency as women but the evidence behind the claim has always been weak which is why this is an important and cleverly designed study that will be widely cited.  Researchers working on gender and TB have been searching for just this parameter for their models, so this study fills an unmet need. Taking place in a HDSS, the investigators obtained hard-to-find denominators for their estimates and stratified by HIV status, a key predictor of health seeking in many settings. It is great to have an estimate of the magnitude of the difference in clinic attendance both for the modellers but also for the funders of different kinds of active case finding efforts.

 This methodology should be emulated in other settings and WHO should recommend this approach to estimation of health seeking as the starting point for all national TB care cascade and patient pathway studies instead of the flawed proxy currently recommended (i.e. use of the entirely hypothetical child fever health seeking question from demographic and health surveys. The use of health seeking for child fever is invalid for TB symptom health seeking behavior and obscures the important gender differences shown by the authors. The authors are giving us a great new way to work in a gender disaggregated way and it would be handy if they provided an SOP to help other DHSS with the linking aspect.

 To be thorough, I offer some critiques:

1. The authors assert that because HIV-negative men are less likely to seek formal public health care during day time hours, that non-passive case finding may be needed to reach this population with TB screening services. However, they do not mention exactly what that would look like. I infer that they are well aware that actually, men are also less likely to participate in generic community TB screening activities (and the South African TB prevalence survey is an example). Therefore the authors were strategically vague as to possible solutions to this challenge.
2. In the abstract the proportion of HIV-negative men seeking any care was (12.7%, 95%CI 11.6–13.8), but in the conclusion, it is given as 15%, which is the correct proportion?
3. The authors refer to HIV-negative men as a “Notoriously Hard To Reach Key Population”, which connotes that repeated efforts have been made to reach men and therefore they obtained notoriety due to multiple failures be reached.  I am not aware of many failed scientifically-driven efforts to reach men with TB in the community. On the contrary, the TB scientific community has simply been negligent in serving the needs of its largest risk group, a group that has been consistently under-served and over-represented for decades if not centuries. It is important to situate the blame for the current situation on public health actors and not the people with TB.
4. The authors identify the absence of night and weekend data as a limitation that would have no impact on the robustness of the estimates because TB screening would not be feasible, but I was not convinced. Men are disproportionately impacted by trauma and they make up the bulk of the admissions for trauma. The statistics probably included labor and delivery even though that service is not particularly conducive to TB screening either. It would not have detracted greatly from the paper to have just conceded that it was a limitation, that the missing data was non-random, and tried to do a bit of sensitivity analysis around it.
5. There is now a rich and informative literature on what South African men with TB want in health care and why they do not seek care in public health spaces. Unfortunately, this important body of work was not as well cited in this manuscript as it could have been. It would have been useful for hypothesis generation and for the discussion. The list of lovely papers and books about TB, health seeking and men is long, and many of the best are focused on South African men:

- 1. *Chikovore J, Pai M, Horton KC, Daftary A, Kumwenda MK, Hart G, et al. Missing men with tuberculosis: the need to address structural influences and implement targeted and multidimensional interventions. BMJ Glob Heal. 2020;5(5):e002255.*
- *2.    Daniels J, Medina-Marino A, Glockner K, Grew E, Ngcelwane N, Kipp A. Masculinity, resources, and retention in care: South African men’s behaviors and experiences while engaged in TB care and treatment. Soc Sci Med. 2021;270:1–15.*
- *3.    Gibbs A, Sikweyiya Y, Jewkes R. “Men value their dignity”: Securing respect and identity construction in urban informal settlements in South Africa. Glob Health Action. 2015;8(1).*
- *4.    Van Heerden A, Msweli S, Van Rooyen H. “Men don’t want things to be seen or known about them”: A mixed-methods study to locate men in a home based counselling and testing programme in KwaZulu-Natal, South Africa. African J AIDS Res. 2015;14(4):353–9.*
- *5.    Reihling H. Affective Health and Masculinities in South Africa [Internet]. London: Routledge; 2020. Available from: https://www.taylorfrancis.com/books/9781000050462*

The authors conclude with a call for further research to understand men’s behavior better, which is fine. Yet this belies the fact there is quite some research on-going on men and TB and/or men and health seeking. One might argue that what is really needed at this juncture is rigorously controlled and well-resourced trials to drive pro-men policy changes to remake the TB service delivery paradigm so that does not devalue or turn off one of its most important client groups.

 [ *I still think the field (not these authors particularly) would do well to try to study how we got here. How did we arrive at a situation where our largest and most consequential risk group has been so under-served? It is kind of flabbergasting how we have collectively dropped the ball and it would be worthwhile to unpack that so we don’t do it again*.]

 In conclusion, this is a methodologically important paper and I hope it is widely replicated.

Is the work clearly and accurately presented and does it cite the current literature?

Yes

If applicable, is the statistical analysis and its interpretation appropriate?

Yes

Are all the source data underlying the results available to ensure full reproducibility?

Yes

Is the study design appropriate and is the work technically sound?

Yes

Are the conclusions drawn adequately supported by the results?

Yes

Are sufficient details of methods and analysis provided to allow replication by others?

Yes

Reviewer Expertise:

TB epidemiology,  stigma and gender

I confirm that I have read this submission and believe that I have an appropriate level of expertise to confirm that it is of an acceptable scientific standard.

## References

[1] : Missing men with tuberculosis: the need to address structural influences and implement targeted and multidimensional interventions. *BMJ Glob Health* .5(5) : 10.1136/bmjgh-2019-002255 10.1136/bmjgh-2019-002255 32371568PMC7223013

[2] : Masculinity, resources, and retention in care: South African men's behaviors and experiences while engaged in TB care and treatment. *Soc Sci Med* .270: 10.1016/j.socscimed.2020.113639 113639 10.1016/j.socscimed.2020.113639 33493956PMC8445063

[3] : 'Men value their dignity': securing respect and identity construction in urban informal settlements in South Africa. *Glob Health Action* .2014;7: 10.3402/gha.v7.23676 23676 10.3402/gha.v7.23676 24717188PMC3982114

[4] : “Men don't want things to be seen or known about them”: A mixed-methods study to locate men in a home based counselling and testing programme in KwaZulu-Natal, South Africa. *African Journal of AIDS Research* .2015;14(4) : 10.2989/16085906.2015.1121881 353-359 10.2989/16085906.2015.1121881

[5] : Affective Health and Masculinities in South Africa. *Routledge* .2020; Reference source
